# The Conundrum of Poor Ovarian Response: From Diagnosis to Treatment

**DOI:** 10.3390/diagnostics10090687

**Published:** 2020-09-11

**Authors:** Polina Giannelou, Mara Simopoulou, Sokratis Grigoriadis, Evangelos Makrakis, Adamantia Kontogeorgi, Agni Pantou, Dionysios Galatis, Theodoros Kalampokas, Panagiotis Bakas, Stamatis Bolaris, Konstantinos Pantos, Konstantinos Sfakianoudis

**Affiliations:** 1 Department of Physiology, Medical School, National and Kapodistrian University of Athens, 75, Mikras Asias, 11527 Athens, Greece; lina.giannelou@gmail.com (P.G.); sokratis-grigoriadis@hotmail.com (S.G.); galatismd@gmail.com (D.G.); 2 Centre for Human Reproduction, Genesis Athens Clinic, 14–16, Papanikoli, 15232 Athens, Greece; agnipantos@gmail.com (A.P.); info@pantos.gr (K.P.); sfakianosc@yahoo.gr (K.S.); 3 Second Department of Obstetrics and Gynecology, Aretaieion Hospital, Medical School, National and Kapodistrian University of Athens, 76, Vasilisis Sofias Avenue, 11528 Athens, Greece; kalamp@yahoo.com (T.K.); p_bakas@yahoo.com (P.B.); 4 Third Department of Obstetrics and Gynecology, Attikon Hospital, Medical School, National and Kapodistrian University of Athens, 1, Rimini, 12462 Haidari-Athens, Greece; evmakrakis@gmail.com (E.M.); adamantia.kondogewrgi@gmail.com (A.K.); 5 Assisted Conception Unit, General-Maternity District Hospital “Elena Venizelou”, Elenas Venizelou Avenue, 11521 Athens, Greece; sbolaris@gmail.com

**Keywords:** poor ovarian response, diagnosis, management, Bologna criteria, Poseidon group, gonadotropin stimulation, adjuvant treatment, second follicular wave, novel approaches

## Abstract

Despite recent striking advances in assisted reproductive technology (ART), poor ovarian response (POR) diagnosis and treatment is still considered challenging. Poor responders constitute a heterogeneous cohort with the common denominator of under-responding to controlled ovarian stimulation. Inevitably, respective success rates are significantly compromised. As POR pathophysiology entails the elusive factor of compromised ovarian function, both diagnosis and management fuel an ongoing heated debate depicted in the literature. From the criteria employed for diagnosis to the plethora of strategies and adjuvant therapies proposed, the conundrum of POR still puzzles the practitioner. What is more, novel treatment approaches from stem cell therapy and platelet-rich plasma intra-ovarian infusion to mitochondrial replacement therapy have emerged, albeit not claiming clinical routine status yet. The complex and time sensitive nature of this subgroup of infertile patients indicates the demand for a consensus on a horizontally accepted definition, diagnosis and subsequent effective treating strategy. This critical review analyzes the standing criteria employed in order to diagnose and aptly categorize POR patients, while it proceeds to critically evaluate current and novel strategies regarding their management. Discrepancies in diagnosis and respective implications are discussed, while the existing diversity in management options highlights the need for individualized management.

## 1. Introduction

The development of assisted reproductive technology (ART) during the last four decades has successfully addressed a plethora of infertile couples. Improving clinical pregnancy rates remains at the top of a researchers’ agenda [1]. Challenges encountered in successfully managing infertile couples are considerable as there is a wide range of etiology entailed. Notably, in vitro fertilization (IVF) still presents with lower success rates for women that fail to adequately respond to controlled ovarian stimulation, also known as “poor responders” [2]. The incidence of poor ovarian response (POR) following ovarian stimulation varies worldwide between 5.6 and 35.1% [3,4]. Over the years, the efficient management of this strictly defined group of patients categorized as POR remains a conundrum [5].

Since the first publication on POR and during the last 37 years there has been an explosion of interest articulated on this topic, reflected through the several hundreds of publications dedicated to identifying physiological pathways, pathogenesis, molecular mechanisms, clinical characteristics, and management options [3,5,6,7,8,9]. Despite the great advances observed in the field, it appears that ongoing controversy fuels a heated debate on various levels from POR diagnosis to treatment [10,11,12]. Presenting these patients with an effective treatment protocol among the many options is based on a timely and precise diagnosis. It is imperative for both aspects to be equally and concurrently addressed, one side taking into consideration the other. The urgent need for a unanimous well-defined classification and treatment following larger and well-established studies is clear [10,11,12].

A plethora of strategies and stimulation protocols have been proposed in the current literature [13]. In the last few decades, a variety of different gonadotropins stimulation protocols, adjuvant therapies and protocols employing a second oocyte retrieval in an individual menstrual cycle have been proposed in an effort to increase the oocyte yield of POR patients [3,14,15]. Choosing the most suitable protocol or treatment strategy for these patients may provide them with a solution addressing a highly time-sensitive matter. Nonetheless, there appears to be no universally accepted strategy for this sensitive patient subgroup [2], and this served as the driver behind this study. Interestingly, in the advent of failing to effectively treat POR and address infertility, their reproductive autonomy right to an offspring that is genetically linked is challenged as oocyte donation presents as the last resort and the next step [16]. Today, the noted patient demand to circumvent these issues and provide alternatives has led to the emergence of innovative approaches such as platelet-rich plasma (PRP), stem cell therapy and mitochondrial replacement therapy (MRT) [17,18,19,20,21]. It is the lack of consensus and full-proof protocols that challenge clinical routine practice and leave clinicians employing more empirical rather than entirely evidence-based treatment options. This, coupled with the risks entailed in the novel approaches emerging—albeit lacking clinical routine practice status yet—underlines this contribution as timely and essential.

This critical review aims to present diagnostic tools and scenarios, while the diverse treatment approaches and current options, in the treatment management of poor responders, are discussed. What is more, this article attempts a critical analysis of the various novel applications recently emerging as candidates in solving the ‘puzzle’ of effective POR treatment and reporting back to the practitioner, while considerations and concerns towards the divergent options on addressing this heterogeneous population are acknowledged.

## 2. Poor Ovarian Response Diagnosis and Patients’ Categorization

Our understanding of human ovarian reserve assumes that the ovary consists of a predefined number of follicles established in utero that are reduced with aging, leading to menopause [22]. The core of the pathophysiology of poor ovarian response is the limited number of follicles responding to FSH [23]. POR is a phenomenon that was firstly linked to advanced maternal age leading to accelerated follicular loss [22]. However, some conditions such as obesity [24] or underlying polymorphisms of a genetic nature may hold the potential to modify the follicular response to exogenous gonadotrophins [25,26,27,28]. Therefore, it has become evident that POR is not attributed to a single cause; the population of POR is heterogeneous and challenging to characterize and our understanding of its pathophysiology is limited.

The first attempt to classify women as poor responder patients was performed by Garcia et al. in 1983 [29]. Numerous underlying mechanisms have been proposed to result in the phenomenon of poor response, from follicular insensitivity to exogenous gonadotrophins, down to even lifestyle and profiling factors such as smoking [30,31]. Additionally, several associations and causative relationships regarding POR have been investigated and reported over the years. These may range from advanced maternal age, to genetic or even iatrogenic factors possibly related to ovarian surgery, pelvic adhesions and endometriosis [4]. The lack of an unanimously and horizontally accepted definition for the term ‘poor ovarian response’ may have caused controversy, albeit several attempts have been noted and certain criteria have prevailed.

In an effort to establish a common consensus for the definition of POR, in 2011 the European Society of Human Reproduction and Embryology (ESHRE) in the annual meeting proposed the ‘Bologna’ criteria [6,32]. According to these criteria, POR women should fulfill at least two out of the following criteria: a) advanced maternal age (above or equal to 40 years) or the presentation of other risk factors for an insufficient response, such as previous ovarian surgery, genetic defects, chemotherapy or autoimmune disorder, b) a former episode of poor ovarian response (oocyte yield of ≤3) following standard COS, and c) a low ovarian reserve with an antral follicle count (AFC) less than 5–7 follicles and serum anti-müllerian hormone (AMH) less than 0.5–1.1 ng/mL. When other criteria are not present, recording at least two incidents of poor ovarian response following COS may serve as adequate justification to define a poor responder patient [6]. The abovementioned criteria constituted an original effort subject to precision systematic analysis in aptly defining patients who respond poorly to COS. A recently published systematic review and meta-analysis reports that the ‘Bologna’ criteria were adopted as inclusion criteria for POR identification in almost half of the interventional trials registered in clinicaltrials.gov since 2011 [3].

Even though recruitment of the ‘Bologna’ criteria in studies still increases, the heated debate in the literature perseveres. In 2014, Ferraretti and Gianaroli reported that “The minimal criteria of the ESHRE consensus are not ‘perfect’ and may need to be revised and implemented” as they highlight the need for new researches to study in depth the effect of any intervention on the clinical prognosis of POR [32]. It appears that, as anticipated, it is the inevitable heterogeneity of the characteristics of poor ovarian responders that established difficulties in implementing the Bologna criteria in clinical practice, compromising the notable effort made by ESHRE in classifying patients of POR [32,33,34].

As the variability regarding the definition of POR is striking, the applicability of the Bologna criteria may require further investigation [34]. Addressing this standing need, a new approach to categorize patients with ‘anticipated’ or ‘not anticipated’ low ovarian response was developed in 2016 to provide a higher resolution image of POR [35,36]. The POSEIDON group (Patient-Oriented Strategies Encompassing IndividualizeD Oocyte Number) suggested a new categorization of ART in patients presenting with a diminished ovarian reserve or unexpected inadequate ovarian response to exogenous gonadotropins in an effort to offer a more treatment-oriented definition [35]. In this form of classification, four subgroups have been proposed based on the following parameters: a) maternal age and the expected aneuploidy rate, b) the results from ovarian reserve biomarkers such as AFC and AMH, and c) ovarian response in previous cycles following ovarian stimulation protocols. Moreover, The POSEIDON group presented a new tool guiding clinicians towards retrieving the required oocyte yield in order to ascertain at least one euploid embryo eligible for embryo transfer [35]. In the authors’ own words “the POSEIDON group aims to offer a practical endpoint to clinicians as it may assist towards establishing a clear line of management in cases of “low prognosis patients” in ART [36].

Fast forward to today, regardless of the definition and the classification of these patients, patients of poor ovarian response present with higher cycle cancellation rates and lower pregnancy rates per embryo transfer with lower cumulative pregnancy rates per initiated cycle than are found in normal responders [3]. The lack of consistency regarding the actual criteria employed by poor responder studies is a reality that merits consideration, especially in light of studies reporting on old versus young poor responders [37]. It is an absolute requirement to proceed by enriching the standing criteria aiming to successfully profile the distinct group of poor responders that share as many similarities as differences. It appears that poor responders may include patients entailing numerous striking differences in profiling. Employing new tools, such as micro RNA analysis, may lead to a more detailed profiling of POR patients. For example, it is demonstrated that altered micro RNA expression in cumulus cells is related to events of poor ovarian response in IVF. A study on micro RNA profiling has highlighted the association between miR-21-5p overexpression and POR, irrespectively of E_2_ levels [38]. Moreover, in another study in which the potential role of micro RNAs in POR was investigated, 23 micro RNAs were found to be upregulated in POR patients. The vast majority of these miRNAs constitute regulators of cell cycle and granulosa cell apoptosis, indicating that granulosa cell apoptosis appears to contribute to POR phenotype [39]. Recruiting micro RNA data in the service of better understanding POR patients is certainly promising and the field merits further investigation. Studies focusing on investigating micro RNA levels and associating them with respective phenotypes in POR may be of value and hold the key for apt profiling. Nonetheless, it appears that we have yet to determine further pathways and move beyond mere associations in order for micro RNA data to be translated to potential biomarkers.

Poor response is a condition encountered in the context of IVF treatment, perhaps implying a pathological condition. However, that may not necessarily be the case as physiological mechanisms may equally be implicated leading to POR, and we need to acknowledge this fact. There is an overlap between pathophysiology and physiology. Ovarian insufficiency brought upon by advanced maternal age includes physiological pathways and mechanisms. The same cannot be claimed for premature ovarian insufficiency implicating pathophysiological pathways [40]. POR patients along with perimenopausal and premature ovarian insufficiency women are all under the umbrella of ovarian insufficiency, sharing similarities, especially those pertaining to manifestation and clinical symptoms. Nonetheless, they are characterized by striking differences as well, mainly pertaining to the etiology extending to the respective pathophysiological pathways and molecular patterns. Surely in the era of precision medicine we need to understand and account for these differences, and we need to acknowledge the basis of the pathological mechanism leading to POR, employing an individualized perspective. Nowadays, it is imperative that we offer treatment not relying on clinical manifestations and the respective clinical observations but treatment that is tailored according to the varying mechanisms and the pathways involved in leading to the same clinical manifestations and the same diagnosis.

## 3. Managing Poor Ovarian Response

### 3.1. Stimulation Protocols

The proposed therapeutic approaches for managing POR patients have been a challenge and a controversial issue for clinicians in the past few years. Cumulative live birth rate per initiated cycle is considered to be the most important outcome in IVF and it is in direct association to oocyte yield following controlled ovarian stimulation (COS) [41]. Consequently, it is pivotal to the treatment strategy to maximize the oocyte yield based on the ovarian reserve of an individual patient during every IVF cycle [6,41].

Various stimulation regimens and interventions have been extensively described in the international literature [5,42]. Some of them report on different gonadotrophin dosages, and others focus on alternative regimens for pituitary suppression [5,43,44]. Inducing ovulation constitutes a milestone during fertility treatment in women of impaired ovarian dynamic.

One of the questions raised pertains to what should be recommended for POR, between GnRH antagonist and GnRH agonist employment. Randomized clinical trials (RCTs) and certain meta-analyses have been conducted to report on the efficacy and safety of GnRH agonist and antagonist protocols. Numerous articles compare stimulation protocols for poor responders indicating superiority or inferiority for the protocols tested. Interestingly, many of these reports fail to address the significant variation existing in pregnancy rates, failing to provide the community with all-inclusive data [45,46,47,48,49]. In October 2019, the ESHRE Reproductive Endocrinology Guideline Group published the ESHRE Guidelines for ovarian stimulation for IVF/ICSI cycles recommending that GnRH antagonists and GnRH agonists are equally recommended and there are no differences in terms of safety and efficacy between these groups for treating POR [50].

In 2017, Lensen et al. published a thorough Cochrane meta-analysis investigating direct gonadotropin dosages for treating POR [51]. In this report, the authors include five RCTs and three groups of comparison emerged. No significant difference was observed comparing gonadotropin dosages of 150 IU vs. 300/450 IU, 300 IU vs. 400/450 IU, and 450 IU vs. 600 IU with respect to live birth and ongoing pregnancy rates. Nonetheless, data indicate that gonadotropin administration at a dose higher than 150 IU results in a higher number of oocytes retrieved in POR patients. However, there is no difference in live birth/ongoing pregnancy rates [50]. Considering the aforementioned, it remains unclear whether an increase in dosage of gonadotropins over 150 IU could enhance IVF outcome [50]. Robust data indicate no beneficial effect of gonadotropin administration at a dose over 300 IU [50]. What is described as a mild or minimal stimulation protocol is defined by employing a lower dose of follicle-stimulating hormone (FSH) or human menopausal gonadotropin (hMG) for a short period of time in a gonadotropin-releasing hormone (GnRH) antagonist co-treatment cycle [52]. Commonly, following a mild protocol in an IVF cycle we expect to retrieve 2–7 oocytes per cycle [5]. In another recent meta-analysis, Jalamudin et al. clearly proposed mild stimulation protocols as the recommended first-line protocol of choice for addressing POR cases indicating an increased live birth rate with minimal oocyte yield [5]. The same report presents mild stimulation protocols as a patient-friendly and cost effective option for POR [5]. The aforementioned advantage of obtaining comparable success rates for POR patients while administrating lower dosages coupled by a lower cost led to the Practice Committee of the American Society of Reproduction Medicine to recommend mild ovarian stimulation protocols for treating POR patients [53].

At this point, it is of interest to note that corifollitropin alfa has been proposed as an alternative option for daily recombinant FSH injections in POR patients undergoing ovarian stimulation in IVF treatment [54,55,56]. Corifollitropin alpha constitutes a hybrid molecule generated by the fusion of human Chorionic Gonadotropin β subunit carboxyl-terminal peptide with human FSH beta chain [57,58]. This molecule is characterized by a slower absorption and a longer plasmatic half-life compared to recombinant FSH while maintaining the same pharmacodynamic profile [59,60,61]. Considering corifollitropin alfa’s pharmacodynamic, it has been voiced that a single injection of corifollitropin alfa could provide the same stimulation outcome as daily recombinant FSH administration, leading to a more patient-friendly stimulation protocol. This approach may alleviate patients’ anxiety levels related to the multiple injections that are typically required when employing recombinant FSH [55]. Data indicate that corifollitropin alfa administration is associated with a higher oocyte yield in women presenting with POR, while some of these studies suggest that corifollitropin alfa may better suit POR patients in comparison to daily FSH administration [54,56,60]. In a recently published systematic review and meta-analysis, the authors provide evidence that corifollitropin alfa administration could be as effective as recombinant FSH for ovarian stimulation in POR patients [56]. However, there is a limited number of well-designed RCTs investigating the efficiency as well as the safety of corifollitropin alfa and for that reason several concerns have been raised by experts in the field [55,56]. Considering the aforementioned, no safe conclusions can be drawn with regard to the use of corifollitropin alfa in clinical practice.

Another heated debate has been noted in the field regarding stimulation protocols employing the administration of letrozole and clomiphene citrate (CC) to enhance the stimulation outcome in POR patients [3].

Letrozole constitutes a selective, non-steroidal third generation aromatase inhibitor [62]. It has been voiced that letrozole administration inhibiting the aromatase enzyme activity reduces estradiol production from androstenedione and testosterone [63]. Data demonstrate that in the early follicular phase, the reduction observed in estradiol synthesis, following letrozole administration, reduces the negative feedback of estrogens in hypothalamus-pituitary levels, promoting endogenous gonadotropins synthesis and secretion [62,63,64]. Considering the aforementioned, it is proposed that letrozole could act as pharmaceutical agent to induce mild stimulation and its use has been investigated in both expected high and low responders [64,65]. Despite the fact that several studies investigating letrozole effectiveness on enhancing stimulation outcomes in expected low responder patients have been so far performed, the results still remain controversial [3]. Administration of letrozole, along with gonadotropins resulted to equal outcomes concerning effectiveness in comparison to high doses of gonadotropins [45]. Opposed to the aforementioned conclusions, it has been further voiced that there is no indication suggesting that letrozole could detrimentally affect outcomes of pregnancy or live birth [66]. In 2017, a meta-analysis by Bechtejew et al., reported that letrozole with FSH in a GnRH antagonist protocol did not differ from conventional ovarian stimulation for IVF/ICSI with regard to clinical pregnancy or oocyte yield [67]. Two years later, the ESHRE guidelines included in their recommendation that the addition of letrozole to gonadotropins in stimulation protocols is probably not recommended for treating POR [50]. Additionally, concerns have been raised regarding possible teratogenesis associated with letrozole [50]. It is essential to acknowledge that future well-designed RCTs are required in order to draw safe conclusions regarding letrozole administration as an adjuvant treatment in POR patients [3].

Administrating clomiphene citrate (CC) is on a similar spectrum with letrozole. Clomiphene citrate is a selective estrogen receptor antagonist and presents with both estrogenic and antioestrogenic actions [68]. At the hypothalamus-pituitary level, CC binds to estrogen receptors, reduces the estrogen-negative feedback and subsequently promotes gonadotropins’ synthesis and secretion [68]. Similarly to letrozole, CC is widely used in clinical practice to induce mild stimulation [69]. With regard to the use of CC for managing POR patients, several studies have been so far performed, including RCTs and systematic reviews and meta-analyses, nonetheless, the results seem to be controversial. It has been reported that COS protocols, employing CC and FSH administration plus GnRH antagonist for POR patients, are correlated with reduced pregnancy rates, number of mature (metaphase II) oocytes obtained, and endometrial thickness compared to COS protocols employing FSH plus GnRH agonist. Moreover, the provided data demonstrated that CC administration failed to increase oocyte yield, or the number of embryos obtained and it further failed to enable decreasing the required dose of gonadotropins for successful COS [70]. However, data provided from systematic reviews and meta-analyses present a different angle. In particular, published data from a meta-analysis, designed to delineate whether administration of CC during a mild controlled stimulation protocol could impact pregnancy rate for POR patients, suggested that COS protocols coupled with CC may result in similar pregnancy outcomes compared to conventional COS protocols employing only gonadotropins [71]. The meta-analysis by Bechtejew et al. reported that CC administration could reduce the required dose of FSH in women with expected POR, providing similar reproductive outcomes with reduced costs [67]. Similar data are also provided from other systematic reviews and meta-analyses [66]. It appears that CC administration in POR patients could decrease the required dose of external gonadotropins in order to achieve a successful COS, without compromising IVF outcomes. Although the above discussed findings may rule in favor of CC employment, it is clear that the quality of evidence suggesting the use of CC administration for POR patients is low, mainly due to the high heterogeneity observed among the studies with regard to several outcome measures [3,66]. In 2019, ESHRE guidelines were issued in an effort to concur on this standing debate, and finally reported that in POR, there are no differences between CC alone, CC in combination with gonadotropins, or gonadotropin stimulation alone in terms of safety and efficacy. Therefore, although the quality of evidence is low, it appears that CC alone or in combination with gonadotrophins, and gonadotropin stimulation alone, are equally recommended in treating poor responders [50].

In summary, the provided data indicate that letrozole should not be considered as an effective agent in order to enhance POR patients’ performance [50]. In contrast, CC alone, CC in combination with gonadotropins or gonadotropin stimulation alone seem to be safe strategies of equal efficiency [50]. However, robust data are still required in order to elucidate the efficiency as well as the appropriate administration scheme, including the appropriate dose, with regard to the use of letrozole and CC protocols for managing POR [50].

### 3.2. Adjuvant Therapies

Besides the use of letrozole and CC, various studies suggest the co-administration of adjuvant therapies including androgen supplements such as testosterone and dehydroepiandrosterone (DHEA) [72,73,74,75,76], growth hormone (GH) [77,78], steroid hormones such as estradiol and progesterone [79,80,81,82], recombinant LH (rLH) [32,83,84], myoinositol [85], and coenzyme Q10 (CoQ10) [86]. Given the wide variety of their diverse mechanisms of action, all the aforementioned adjuvant therapies have been proposed to improve pregnancy outcomes by targeting various aspects, from improving follicular development and oocyte maturation to enhancing embryo quality and endometrial receptivity [3].

Several studies indicate that androgen supplements such as testosterone and dehydroepiandrosterone (DHEA) could enhance POR patients’ performance [72,73,74,75,76]. In 2018, a meta-analysis highlighted that androgens may play a key role in folliculogenesis by increasing ovarian responsiveness to gonadotropins, suggesting that maybe there is a positive impact of androgens on the folliculogenesis process, although the exact molecular pathway is still unclear [87].

On the other hand, it is reported that low-dose GH supplementation could increase the success rates of patients presenting with POR undergoing IVF treatment [77,78]. It has been proposed that GH could increase the sensitivity of granulosa cells to gonadotropins via the insulin-like growth factor 1 (IGF-1) pathway, promoting follicular recruitment [88]. The data provided indicate that GH supplementation could increase the number of retrieved oocytes and could reduce the duration of ovarian stimulation required prior to oocyte retrieval [88]. However, the quality of evidence supporting the use of GH for managing POR still remains poor and no safe conclusions can be reached with regard to the use of GH in clinical routine practice [3].

Moreover, sex steroid hormones, including estradiol and progesterone [79,80,81,82], have been investigated in the literature as possible adjuvant therapies for treating POR. It is demonstrated that estrogen and progesterone administration could improve stimulation outcomes as well as endometrial receptivity. However, its role in managing POR remains unclear [3].

Recombinant LH (rLH) is another adjuvant therapy proposed as an effective reagent towards enhancing POR patients’ IVF outcomes [3]. However, robust data indicate that rLH administration is not correlated with improved COS performance, suggesting that endogenous serum LH levels adequately support follicular ovarian steroidogenesis. Moreover, the optimal daily dose and supplementation timing of rLH are still under consideration [32,83,84].

With regard to myoinositol, corifollitropin and CoQ10 administration, data provided are still inconclusive. Myoinositol has been shown to be beneficial with regard to oocyte quality in women presenting with polycystic ovary syndrome. However the beneficial effect of co-treatment with myoinositol for POR patients has not been proven so far [85]. In 2018, an RCT reported that pretreatment with coenzyme Q10 (CoQ10) may improve ovarian response and embryo quality in women of poor prognosis during IVF [86].

Early this year, a systematic review and network meta-analysis was conducted in order to provide robust evidence with regard to the efficiency of different adjuvant treatment strategies in managing POR patients undergoing IVF [3]. The data provided indicate that COS protocols supplemented with adjuvants including DHEA, CoQ10 and GH are correlated with higher chances of achieving a pregnancy as well as with a lower dosage of gonadotrophins for follicular recruitment [3]. However, in the same study, the authors highlighted the fact that, despite the plethora of publications, it is evident that no safe conclusions can be reached yet with regard to the use of adjuvant therapies for managing POR.

To conclude, the use of adjuvants has yet to be showcased as a unanimously accepted treatment for POR due to their ambiguous efficiency. Data provided by large and well-designed RCTs is of paramount importance in order to properly investigate the parameters related to adjuvant therapies, including the optimal protocol of administration as well as safety issues. Taking into account the heightened heterogeneity of POR patients, the effectiveness of adjuvant treatments should be thoroughly justified as clinical individualized treatment requires foolproof data.

### 3.3. Natural Cycle for Managing Poor Responders

In ART, a natural cycle entails the process of vaginal oocyte retrieval following identification of the mature Graafian follicle during the follicular phase of a spontaneous menstrual cycle. The yielded oocyte is subsequently fertilized while the natural cycle concludes with an embryo transfer [52]. The heightened risk of cancellation entailed during a natural cycle prompted clinicians to explore further options that may enable an enhanced number of yielded oocytes, while ascertaining a minimized risk. The concept of a modified natural cycle emerged, thus the idea of performing strictly natural cycles was abandoned [89]. The minimally invasive nature of this procedure and the absence of hormonal treatment were positively acknowledged, rendering the natural cycle a viable alternative [89]. Numerous studies in the literature suggest natural and modified natural cycles to treat POR. In two RCTs where the authors compare the outcomes between the natural cycles of IVF and micro-dose flare and high-dose antagonist protocol, no difference in the data of clinical pregnancies and live birth rate were indicated [90,91]. Nonetheless, a thorough comprehensive review of the literature failed to showcase high-quality RCTs, supporting that a modified natural cycle or natural cycle IVF in poor responders is a valid or optimal option for POR [50]. From one point of view, conventional controlled ovarian stimulation employing higher dosages of gonadotrophins is frequently associated with a variety of side effects, higher costs and an added psychological and financial strain following futile attempts [3,92,93]. On the other hand, the efficacy of natural cycle IVF for POR may be disappointing and probably fails to ascertain recommendation status on the grounds of the high cancellation rates due to the anticipated low oocyte yield and the scarcity of well-designed RCT_S_ [3,50].

### 3.4. Employing the Second Follicular Wave

It has been consistently and successfully argued in published data that gonadotrophin administration fails to compensate for the absence of follicles in the ovary, therefore COS in poor responders cannot increase the oocyte yield, or IVF success rate. Interestingly, COS may provide added benefit in the context of exploiting multiple follicular waves within a single menstrual cycle. In the last few years, a novel pattern of folliculogenesis has been showcased according to the observation of subsequent follicular waves following the known one observed during the follicular phase [94]. The current data have raised considerations disputing the notion of single folliculogenesis, while introducing the concept of two or even three further such phases [95].

The idea of multiple waves of folliculogenesis was initially documented in animal models, and the hypothesis was further investigated in humans [96]. This led to its introduction in ART, in an effort to ascertain the maximum oocyte yield during retrieval from both the luteal and follicular phase, during an individual menstrual cycle. A novel clinical practice has emerged by employing ovarian stimulation protocols within the luteal phase, succeeding follicular stimulation and oocyte retrieval in an individual menstrual cycle [97]. Over the past few years, researchers explored the new strategy of stimulation in the luteal phase both independently and in combination with standard stimulation protocols, introducing to the clinical field what is described as the protocol of double stimulation (DuoStim) [98,99,100]. As a result, DuoStim consists of an ordinary follicular phase stimulation (FPS) in combination with luteal phase stimulation (LPS) [15,101]. This approach has been associated with increased chances of a pregnancy being ensued. Triggering the ovarian reserve to its maximum potential inevitably resulted in a higher oocyte yield, prompting clinicians to investigate and produce innovative stimulation protocols particularly targeting POR patients [97,98]. Experts in the field of IVF acknowledge DuoStim as an efficient strategy for managing POR, showcasing it as an innovative method for retrieving oocytes during the luteal phase [98].

The very first report in the literature referring to the DuoStim protocol was published by Kuang and colleagues, who proposed the Shanghai protocol [97]. Data presented in the study of Kuang et al. indicate that COS conducted in both FPS and LPS could enhance oocyte yield and subsequently the number of generated embryos, improving the IVF success rate [97]. Since then, several studies were conducted investigating the effectiveness of different DuoStim protocols employing different COS strategies [15,99,101]. A systematic review and meta-analysis, published by Sfakianoudis and colleagues, demonstrated that DuoStim approaches could effectively increase oocyte yield in the same menstrual cycle, and this is a significant outcome considering the time-sensitive nature of POR patients [15]. However, the studies employing the DuoStim approach were characterized by significant heterogeneity, especially with regard to the COS protocol employed [15]. Moreover, the total quality of evidence is poor, considering the lack of RCTs or even large prospective studies evaluating the efficiency of DuoStim [15]. As a result, several questions, with regard to the efficiency and the safety of DuoStim, should be answered prior to delineating the proper requirements and considering DuoStim as a truly effective approach. Firstly, the COS protocol presenting with the best efficiency should be defined. Moreover, it is of pivotal importance to collect robust data indicating that oocytes, and subsequently embryos, originating from the luteal phase of the menstrual cycle are of equal quality and of equal dynamic when compared to those that originate from the follicular phase. Last but not least, it is of high significance to determine the exact POR population who will benefit from the DuoStim approach.

Interestingly, it has been proposed that double oocyte retrieval could be performed in natural IVF cycles without employing COS. In 2018, Sfakianoudis et al. first suggested the term LuPOR for Luteal Phase Oocyte Retrieval [98]. The use of LuPOR in natural cycles was introduced, aiming to report on the potential of this approach in benefitting the special subgroup of poor responders [98]. The results of this study propose that more embryos could be obtained when performing LuPOR in combination with conventional retrieval during the follicular phase [98]. It is proposed that the natural cycle LuPOR treatment strategy coupled with accumulating embryos through multiple natural cycles may lead to new era in managing POR patients [102]. Despite the fact that the LuPOR approach is of great scientific interest considering that the need for COS is circumvented, more data referring to the efficiency, the safety, as well as to the cost-effectiveness of this proposed management-scenario are required.

### 3.5. Oocyte and Embryo Banking “the Accumulation Scenario”

The great advances noted, with regard to oocyte and embryo cryopreservation, since the introduction of vitrification in clinical routine practice, have led the scientific community to investigate a new strategy towards efficiently managing POR patients, namely the “accumulation scenario”. The aim of this strategy is to create a “bank” of frozen oocytes or/and embryos by accumulating them following a number of COS cycles or even via natural or modified natural cycles [103,104,105,106]. The rationale behind this approach, for managing POR patients, is that the IVF success rate could improve, and the drop-out rate could decrease if the number of available embryos for transfer is increased, mimicking a “normoresponder” status.

Regarding oocyte accumulation, published data demonstrate that this could be an effective approach towards enhancing IVF outcomes regarding POR patients [103,105,106]. These data suggest that oocyte accumulation by vitrification for POR significantly reduces patients’ drop-out rates and IVF cancellation rates, as well as significantly increasing the live birth rate per intention-to-treat patient [105]. In a more recent retrospective study, Datta et al. proposed an accumulation of embryos through multiple modified natural cycles that might give the opportunity of better selection and transfer of multiple embryos, leading to improved pregnancy outcomes in POR [102]. Opting for this strategy, the vast majority of women with a reproductive history of three repeated cycles may have at least one embryo for transfer, overcoming the high risk of cycle cancellation associated with a single natural cycle [102].

Despite these interesting outcomes, there are no robust data indicating the efficiency and safety of the “accumulation scenario” regarding both embryos and oocytes. Moreover, significant questions, such as “which is the appropriate number of oocytes and embryos that should be accumulated prior to embryo transfer?” and “which is the best strategy for accumulation”, remain to be answered. Large prospective studies or even RCTs are required to provide robust data in order to verify the efficiency and safety of the “accumulation scenario” approach. Table 1 presents an outline of all current strategies for addressing POR and their recommendation status based on published data.

### 3.6. Novel Approaches in Addressing Poor Ovarian Response

A comprehensive literature review clearly indicates that a well-established and unanimously accepted treatment strategy for POR has not been established yet. This may serve as proof that standing protocols and current approaches may not even present with sufficient effectiveness when dealing with the highly perplex situation of ovarian insufficiency that entails a wide spectrum of pathophysiological conditions. This reality, coupled with the noted patient demand to exhaust all possible options prior to resorting to oocyte donation, serves as the driver for the exploration, design and—to some extent, premature—application of innovative protocols.

In the last few years, studies have focused on exploring novel approaches to address POR in the context of restoring the jeopardized ovarian micro-environment observed in POR patients [107]. Experts attempted the restoration of the ovarian environment, employing factors of known regenerative potential such as stem cells, isolated growth factors or Platelet Rich Plasma (PRP) [19,108]. The first published report exploring the value of stem cells in reproductive medicine was in 1996, presenting patients with poor ovarian insufficiency who went through bone marrow-derived stem cells (BMDSC) transplantation, enabling natural conception [109]. The significant success of stem cell therapy during the last years renders it an option of value in the research field of reproductive sciences. In 2018, a systematic review by Fazeli et al., showed promising results with more than of 11,400 published studies investigating the potential use of stem cell as an effective therapy for several conditions leading to infertility [110]. In the same year, Herraiz and colleagues brought to our attention a new wave of treatment for POR patients by showcasing the potential of ovarian rejuvenation employing stem cell therapy [18,111]. In their study, the authors highlight their interesting results following autologous stem cell ovarian transplant (ASCOT) on the ovarian reserve of POR patients of highly poor prognosis, indicating promising outcomes [18,111]. As it has been reported, the ultimate aim of stem cell therapy is to address age-related ovarian senescence as indicated in women of POR, women with severe diminished ovarian reserve (DOR) and women with premature ovarian insufficiency (POI). Data originated from several studies showcasing encouraging results, indicating the therapeutic potential of stem cells for ovarian rejuvenation [18,19]. However, several concerns related to the effectiveness as well as to the safety of stem cell transplantation for ovarian rejuvenation still stand, hence this approach retains its experimental stage status [112]. At this point, robust data provided from large RCTs and cost-effectiveness studies are required in order for application to extend to clinical routine status.

Platelet-rich plasma is a novel treatment option showcased in various fields that appears in the latest published literature. PRP is comprised of crucial factors identified in repair mechanisms instigating fundamental physiological procedures in healthy tissues [113]. Animal studies in the context of reproductive medicine have described that the intra-ovarian infusion of PRP improves the developmental dynamics of primordial and primary preantral follicles [114]. Moreover, similar studies employing animal models resulted in increased follicular development and a reduction in apoptotic events [115,116]. In 2018, preliminary data on the application of PRP regarding restoration of ovarian function in humans was introduced by two groups of scientists, Sfakianoudis et al. and Sills et al. [108,113,117,118]. For perimenopausal and prematurely menopausal IVF patients of poor ovarian reserve, the reported results showed restoration of ovarian function along with restoration of the menstrual cycle post treatment [108]. On the other hand, data on poor responder patients similarly indicate an impressive hormonal improvement, improved oocyte yield, clinical pregnancies and subsequent live births in a short time interval post PRP treatment [117,119]. Two recent published studies provide data in regard to PRP’s effectiveness in addressing POR, indicating that PRP may be an effective solution towards enhancing IVF/ICSI outcomes for both young and older POR patients [17,120]. However, both of these studies present limitations that are mainly attributed to their observational nature. The use of PRP seems to be a more patient-friendly approach in comparison to stem cell transplantation simply on the grounds of invasiveness. However, there is lack of strong evidence regarding the efficiency of either of these approaches. As previously described for stem cells, PRP is similarly considered to be an experimental approach. Several parameters related to PRP’s safety as well as to the protocol of PRP administration remain to be elucidated [17]. In the context of ART, and mainly driven by patient demand, clinical application of experimental approaches in order to address infertility may become an option [121]. For that reason, it is of paramount importance to meticulously investigate innovative approaches prior to proceeding with routine clinical practice [17].

From a different perspective and considering the fact that one of the defining characteristics noted in POR patients is advanced age, clinicians should focus not only on the number of the yielded oocytes but also on their quality, which will define the dynamics of the subsequent embryos. Mitochondrial dysfunction has been proposed as a parameter that could majorly dictate the quality of the oocyte [122]. Deletions and mutations of the mitochondrial DNA—accumulated during the course of life—constitute the culprits of impaired mitochondrial function [122]. Taking into consideration the pivotal role of mitochondria in controlling energy demands of oocytes, along with the fact that meiosis is a highly energy-dependent event, mitochondrial DNA damage and subsequent deterioration of energy production may also affect critical cellular functions [123,124,125]. In view of these observations, a hypothesis was formed to employ mitochondria from a young healthy donor in order to enhance oocytes’ reproductive competence and provide a better environment during the early developmental stages of the pre-implantation embryo [126,127]. This concept, principally introduced by Cohen and employing ooplasmic transfer, resulted in reports of clinical pregnancy and live birth, until 2001 when the Food and Drug Administration (FDA)decided to ban its application, due to ethical and medical concerns [128]. Recently, a novel aspect of this technique was introduced in the literature. AUGMENT described the Autologous Germline Mitochondrial Energy Transfer (AUGMENT). Their protocol involves isolation of mitochondria from the patient’s oogonial stem cells (OSCs), processed and injected into the patient’s own oocytes during ICSI [129,130,131]. The respective results failed to show an effectiveness through this novel technique, suggesting that it may be possible that the autologous mitochondrial transfer is suitable for a specific population that still remains to be identified. On the other hand, heterologous mitochondrial transfer, known as mitochondrial replacement therapy (MRT), recently emerged with four available protocols, namely, the pronuclear transfer (PNT), the polar body transfer (PBT), the maternal spindle transfer (MST) and the germinal vesicle transfer (GVT) [20,132]. This technique has been employed on the grounds of avoiding transmission of mitochondrial diseases [133,134]. In an effort to recruit mitochondria in the service of POR patients, employment of this technique was suggested to improve IVF outcomes. This has raised considerable ethical concerns in the ART world. The European Society of Human Reproduction and Embryology (ESHRE) issued a formal thesis conveying strong discouragement and disapproval regarding the technique’s application for POR patients. Until now, there has been a lack of robust or even adequate data indicating the safety and effectiveness of the method, which currently retains its experimental status.

Cutting-edge basic research performed in animal models has provided promising data, primarily focusing on the differentiation of primordial oocytes or embryonic stem cells to oocytes able to be fertilized. Despite the distant futuristic approach of employing bioengineering techniques to treat infertile patients, its promising nature regarding the improvement of the therapeutic options offered in ovarian insufficiency patients cannot go unmentioned [135]. These innovative options raise hope, and enrich our options extending to the treatment of POR. Nonetheless, rigorous studies should be performed on safety and efficiency along with addressing the several bioethical points emerging in the prospect of application. Large RCTs and new data should be reported in order to investigate their clinically-effective application. Table 2 presents a summary of data regarding novel approaches suggested for addressing POR, reporting on the type of strategy, invasiveness, mechanisms of action, outcomes and adverse effects.

## 4. Discussion

The international community of IVF physicians and embryologists continually expresses a piqued interest in POR patients and their respective management. This is reflected in the explosion of awareness and concern noted in the published data. Research focuses on all angles investigated, from the pathophysiology of poor response, to molecular delineation in helping us understand the pathways involved, to the definition of apt classification criteria that should be adopted for a precise diagnosis, leading to individualized treatment and novel techniques. Despite all of the efforts and the overwhelming amount of data published, the lack of consistency regarding the actual criteria employed by poor responder studies is a reality that merits consideration. Both the Bologna criteria and those proposed by the POSEIDON group employ several factors that may be used on a singular basis or in combination, serving as categorization factors for such patients. However, the universally acknowledged heterogeneity of this substantial subset of women of POR undergoing IVF, establishes difficulties in applying any of these chosen criteria in a catholic manner. What merits our attention is that although RCTs are initiated and the effectiveness of treatment protocols is thoroughly investigated, we have not yet delineated the true identity of these patients.

It appears that poor responders may include patients entailing numerous striking differences in profiling. Factors such as genetic polymorphisms or obesity may serve as leading factors for poor ovarian response at all ages [136,137,138]. On the other hand, women of advanced age driven by socioeconomic or other issues to delay parenthood, may face diminished ovarian response as well. In the era of personalized medicine, it may be of value to employ tools for detailed profiling in order to distinguish between the biological and the chronological age of these women [139]. Recent studies, analyzing the molecular profile of these women, present interesting results as they reveal an underlying differentiation in their microRNAs [38]. Highlighting these findings and bearing in mind the pathophysiological profile of the POR phenomenon, it may be high time to revise, enrich and update the existing diagnostic tools.

In light of the evidently inadequate current criteria and existing diagnostic tools, a foolproof strategy in addressing these patients remains elusive and subject to empirical approaches [2]. It should be voiced that from the early days of the Assisted Reproduction era, IVF techniques were conceptualized and developed serving the concept of personalized approaches for addressing infertile patients individually. Over the years, as dictated by lifestyle and reorganization of socioeconomical priorities, childbearing has been postponed, introducing new essential parameters when navigating through fertility treatments due to the factor of aging [140]. There is a noted progressive increase in the number of patients requesting ART treatment. This may have contributed to jeopardizing the level of personalization of services when treating a patient. Despite the positive pregnancy outcomes that the universal stimulation protocols have resulted in, one should not fail to notice the equally large percentage of patients for whom these protocols have failed. As a result, employing established and widely acknowledged and applied protocols may be proven to be untrustworthy and perhaps unreliable when targeting certain subgroups of infertile patients with explicit demands. The POR group is a deafening example, showcasing this trend in clinical practice. In the era of precision medicine, it is a paradox that IVF—being one of the first fields embracing and promoting precision medicine practice—is now challenged by it. It is apparent that something is missing when it comes to successfully managing women of reduced ovarian response. Could it be the lack of a consensus? Could it be the extensive variation and heterogeneity evident in poor responders? Could it be that we are still struggling to delineate molecular pathways and understand the plethora of mechanisms involved? The answer still eludes us.

In this comprehensive literature review, we present past, present and future options available to clinicians in order to handle this challenging task. Past and present treatment options employ low or high doses of stimulation protocols, adjuvant treatment therapies, modified natural or natural cycles and strategies such as embryo banking. Novel techniques such as stem cell therapy, PRP and MRT have been presented in the literature for treating poor responders. These novel approaches may or may not provide future solutions to ovarian insufficiency. All these options may lead to confusion and overwhelm patients. This fact, coupled with the high heterogeneity and lack of apt profiling, may lead to ineffective management. Our obligation is firstly to accurately diagnose these patients, taking into account not the clinical manifestations alone, but the etiology and defective pathways leading to this condition. We may need to ask ourselves “is the term Poor responders enough? Is it adequate in describing them? Could it be coupled by a further characterization on that could help us more precisely describe them?” Having secured apt profiling, we need to identify the most efficient protocol. Future studies should employ a trail of methodology and perhaps focus on the development of an algorithm leading to accurate profiling, diagnosis and effective treatment. New, well-established RCTs will provide the data required to fulfil this difficult and noble goal for a patient-centered practice.

What served as the incentive for this work is that fact that, when critically analyzing the studies, and investigating the various protocols and options, it becomes apparent that they lack consistency. In order to properly report on data, specific tools should be employed; if this is not a prerequisite, we are led to contradictions. Further to that, when evaluating safety and efficacy, we need to examine all aspects, not only pregnancy rates, but also embryo quality, side effects and cost effectiveness. Additionally, since time is of the essence and these patients commonly need to undergo multiple cycles, the element of time should also be accounted for in studies. Taking into consideration the sensitive nature of this group and the valuable oocytes retrieved in these IVF cycles, we may further have to revise laboratory protocols as well. Embryologists may choose to exclusively perform ICSI in these cycles in order to keep high fertilization rates [141]. Moreover, in this new era of artificial intelligence in IVF laboratories, time-lapse technology, when possible, may provide important information on the development of these embryos [142,143]. Further to that, as poor responders mainly refer to women of advanced maternal age and previous failed attempts, we may consider employing preimplantation genetic testing for aneuploidy to ensure the chromosomal integrity of the embryos prior to embryo transfer [144]. All these suggestions are certainly subject to examination and hence future studies may provide us with the final verdict as to the benefit their application may convey. Adoption of these practices—when deemed necessary—may lead to an integrated strategy in the battle of POR.

According to recent studies and the unanimous recommendations from scientific communities, low gonadotropin dosages and mild stimulation protocols may provide a more patient-friendly approach. This approach, in combination with new strategies such as LuPOR and DuoStim, may hold the key to an improved management of this sensitive group of infertile patients. Novel options may be constantly emerging, although extreme caution should be exercised as they may be prematurely introduced, lacking the prerequisite platform of solid and well-designed studies employing carefully chosen inclusion and exclusion criteria. Further well-designed basic research studies, enriching the literature, are required in setting the basis for the future establishment of the most effective therapeutic strategy. Nonetheless, perhaps the answer may not be reaching for the “holy-grail” of protocols, but rather in efficiently profiling the poor responder and, respectively, treating them. Pursuing a tailored approach based on patients’ unique characteristics constitutes a more sophisticated policy than embarking on the mass application of therapeutic trends that lack individuality.

## Figures and Tables

**Table 1:** Recommendation status regarding clinical application of current strategies employed for addressing poor ovarian response according to published data.

Strategies	Recommendation Status	Source
Controlled Ovarian Stimulation		
GnRH antagonist	Recommended	Guidelines [50]
GnRH agonist	Recommended	Guidelines [50]
Gonadotropin dose over than 150 IU	Unclear	Guidelines [50]
Gonadotropin dose over than 300 IU	Not recommended	Guidelines [50]
*Adjuvant therapies*	*Evidence*	*Source*
Letrozole	Not recommended	Guidelines [50]
Clomiphene citrate	Recommended	Guidelines [50]
Growth Hormone (GH)	May be Recommended	Meta-analysis [3]
Dehydroepiandrosterone (DHEA)	May be Recommended	Meta-analysis [3]
Coenzyme Q10 (CoQ10)	May be Recommended	Meta-analysis [3]
*Natural Cycle*	Unclear	More studies are needed
*DuoStim approach*	May be Recommended	Meta-analysis [15]
*LuPOR approach*	Unclear	More studies are needed
*Oocyte and Embryo banking*	Unclear	More studies are needed

When guidelines have been published, the status presents as “recommended” or “not recommended”. When data is sourced from systematic reviews and meta-analyses, recommendation status lacks the conclusiveness of guidelines yet allows for the classification “may” or “may not” be recommended. When data is sourced from any other type of studies, the recommended status remains “unclear” as more studies are required to reach a consensus on the effectiveness of clinical application before ascertaining the recommendation status.

**Table 2:** Data regarding novel strategies that have been suggested for addressing poor ovarian response.

Novel Approach	Type	Invasiveness	Function	Published Data	Outcomes	Adverse Effect
Platelet-Rich Plasma Intraovarian Infusion	Autologous	Minimal	Restore ovarian niche	Pilot studies	May increase number of oocyte yield, number of embryos, live birth rate	No adverse effect reported, long-term follow up required
Autologous stem cell ovarian transplantation	Autologous	Minimal to high	Restore ovarian niche	Pilot studies	May increase oocyte yield, number of embryos obtained	No adverse effect reported, long-term follow up required
Mitochondrial replacement therapy	Autologous or heterologous	High	Restore oocyte quality	Small observational studies	May increase pregnancy rate	Unknown adverse effects, several considerations
